# Supplementary material for: How accurate are existing land cover maps for agriculture in Sub-Saharan Africa?
Source: Sci Data. 2024 May 10;11:486. doi: 10.1038/s41597-024-03306-z (PMC11087537; doi:10.1038/s41597-024-03306-z)
Supplement: Supplementary file 1 — Supplement 1 [file 41597_2024_3306_MOESM1_ESM.pdf]

Supplementary File 1

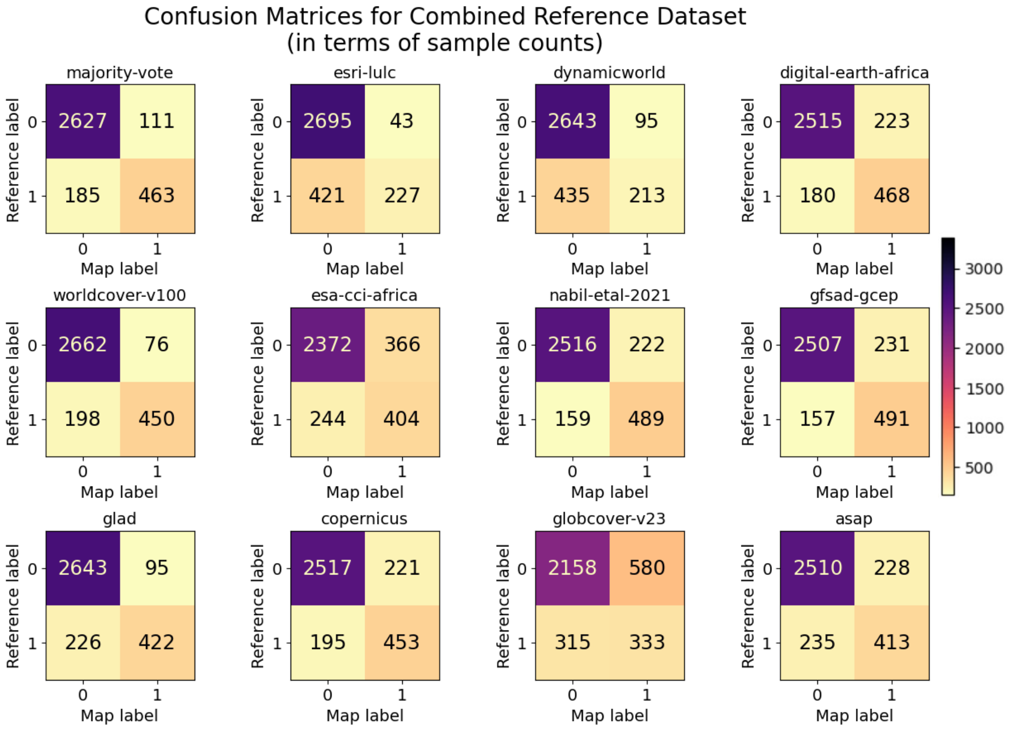

Figure 1. Unnormalized confusion matrix, expressed in terms of sample counts, for each map for the combined reference datasets.

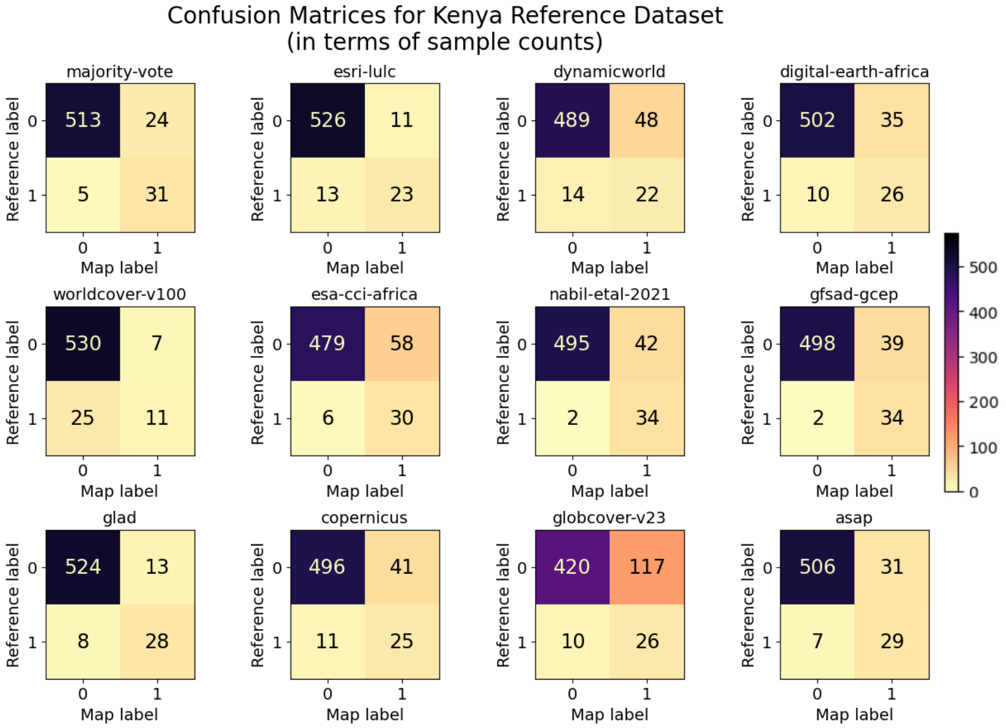

Figure 2. Unnormalized confusion matrix, expressed in terms of sample counts, for the Kenya reference dataset.

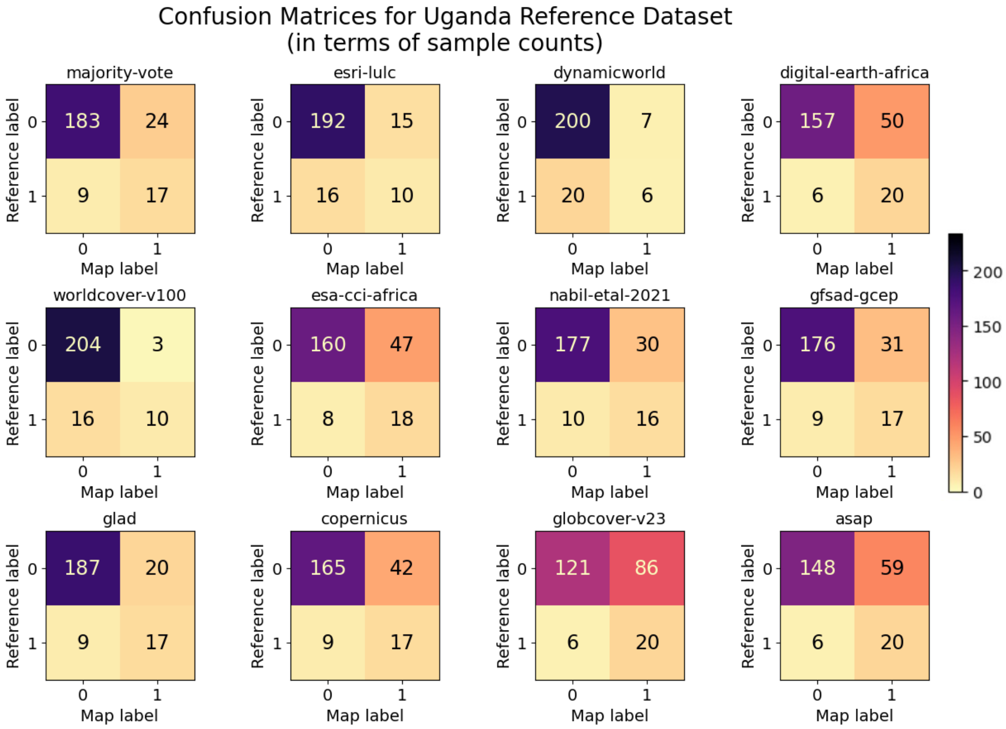

Figure 3. Unnormalized confusion matrix, expressed in terms of sample counts, for the Uganda reference dataset.

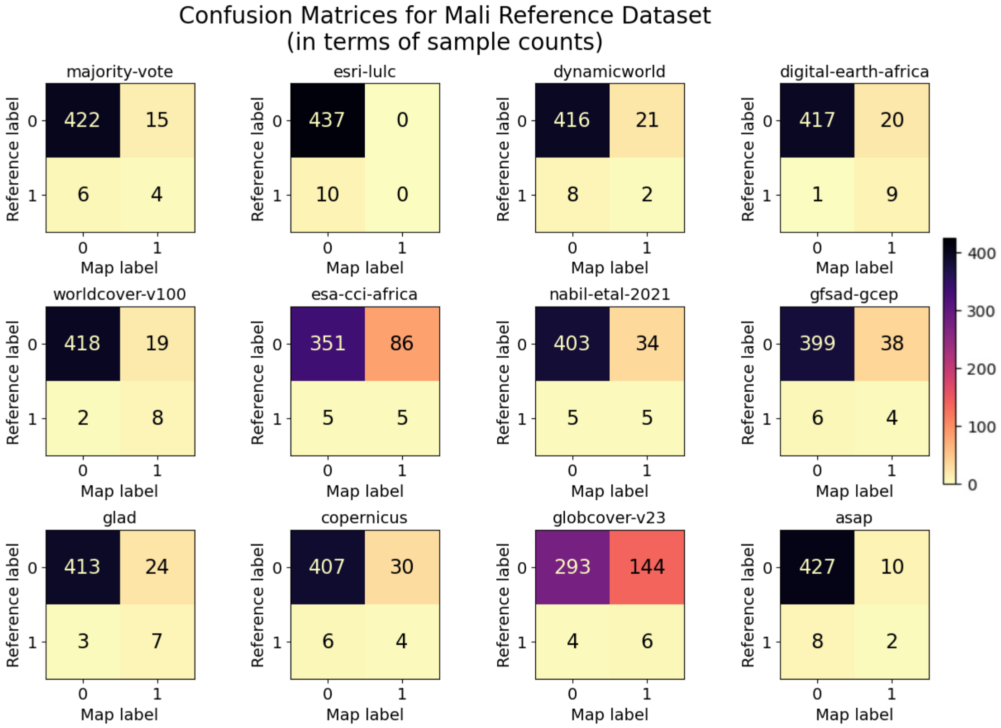

Figure 4. Unnormalized confusion matrix, expressed in terms of sample counts, for the Mali reference dataset.

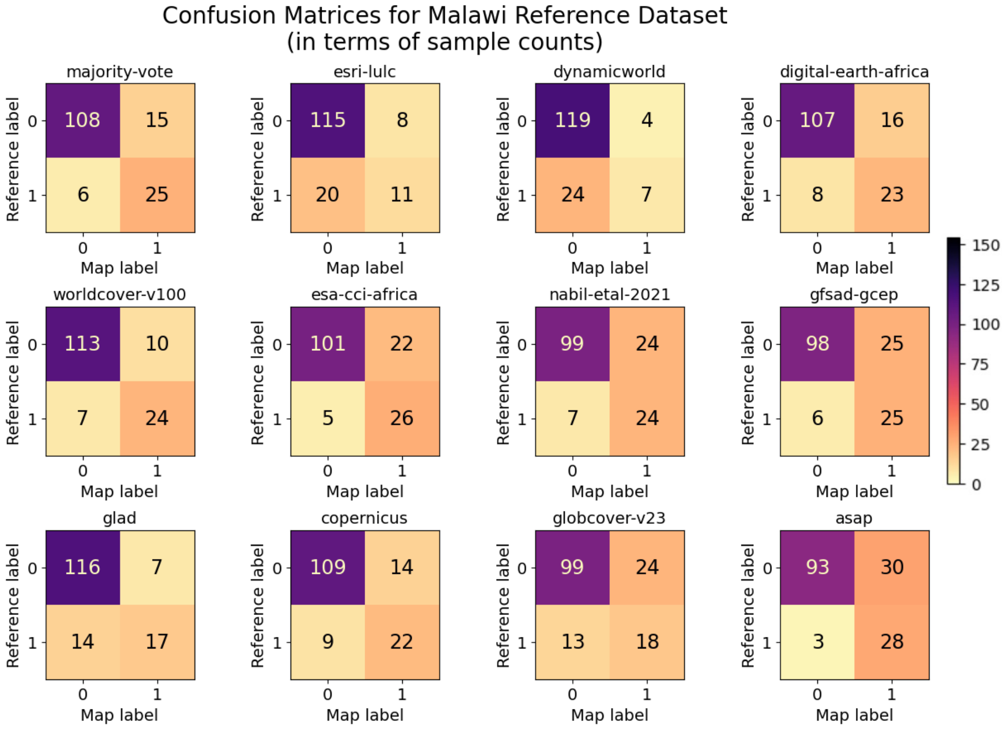

Figure 5. Unnormalized confusion matrix, expressed in terms of sample counts, for the Malawi reference dataset.

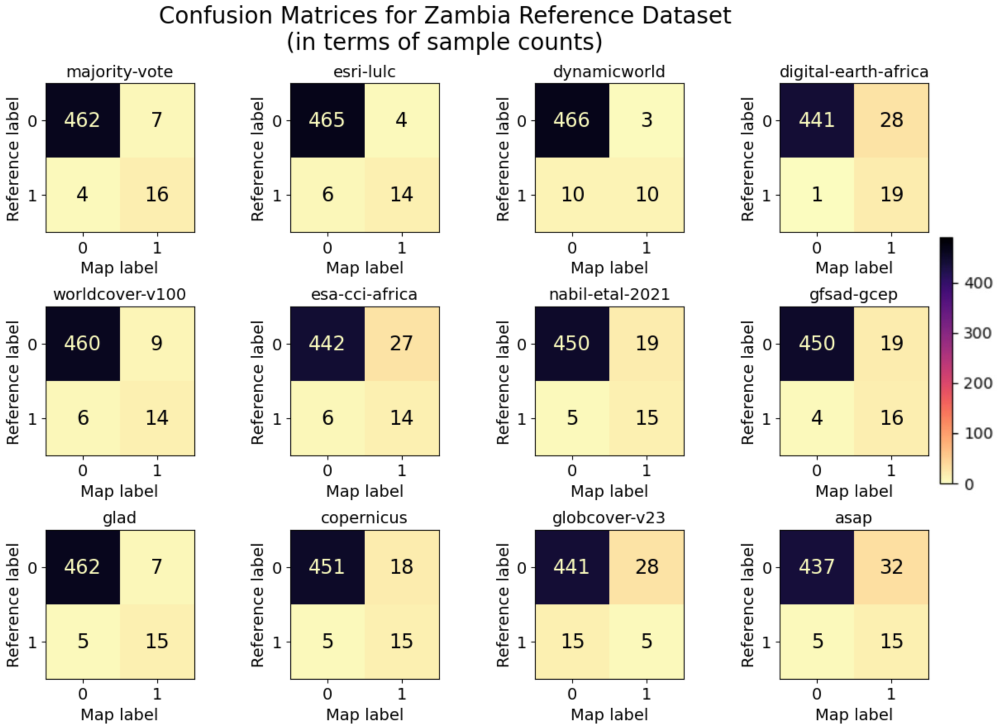

Figure 6. Unnormalized confusion matrix, expressed in terms of sample counts, for the Zambia reference dataset.

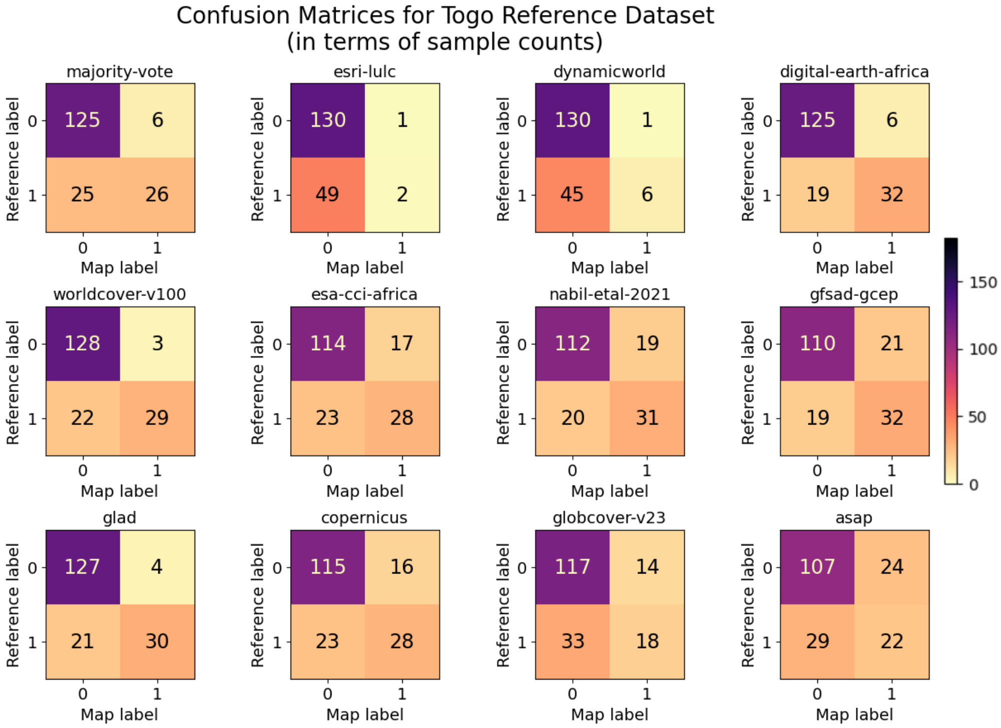

Figure 7. Unnormalized confusion matrix, expressed in terms of sample counts, for the Togo reference dataset.

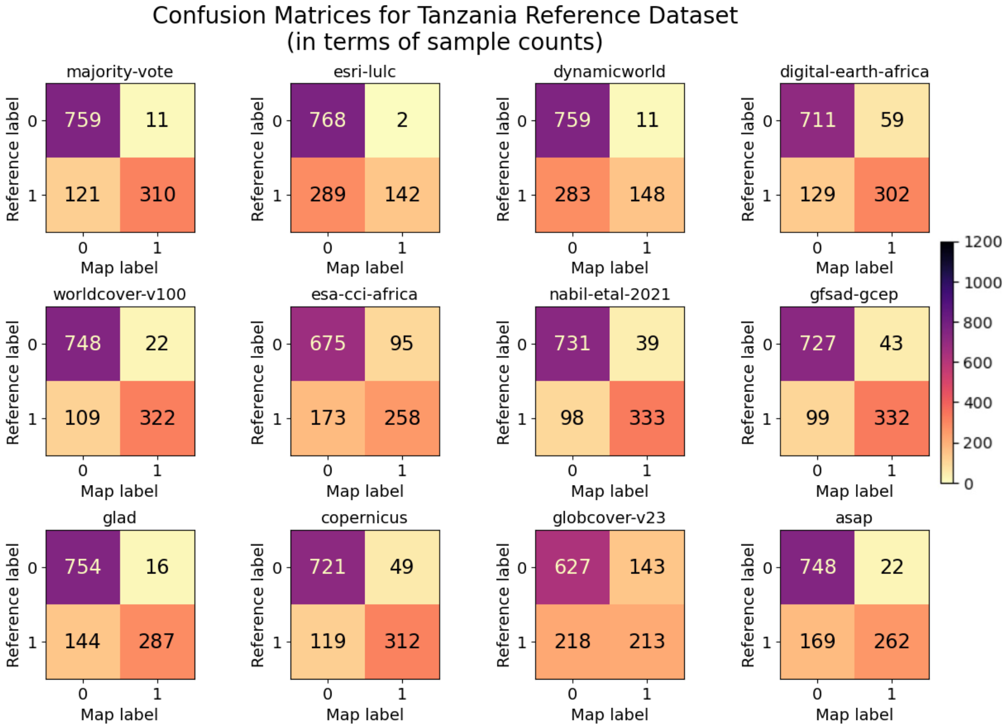

Figure 8. Unnormalized confusion matrix, expressed in terms of sample counts, for the Tanzania reference dataset.

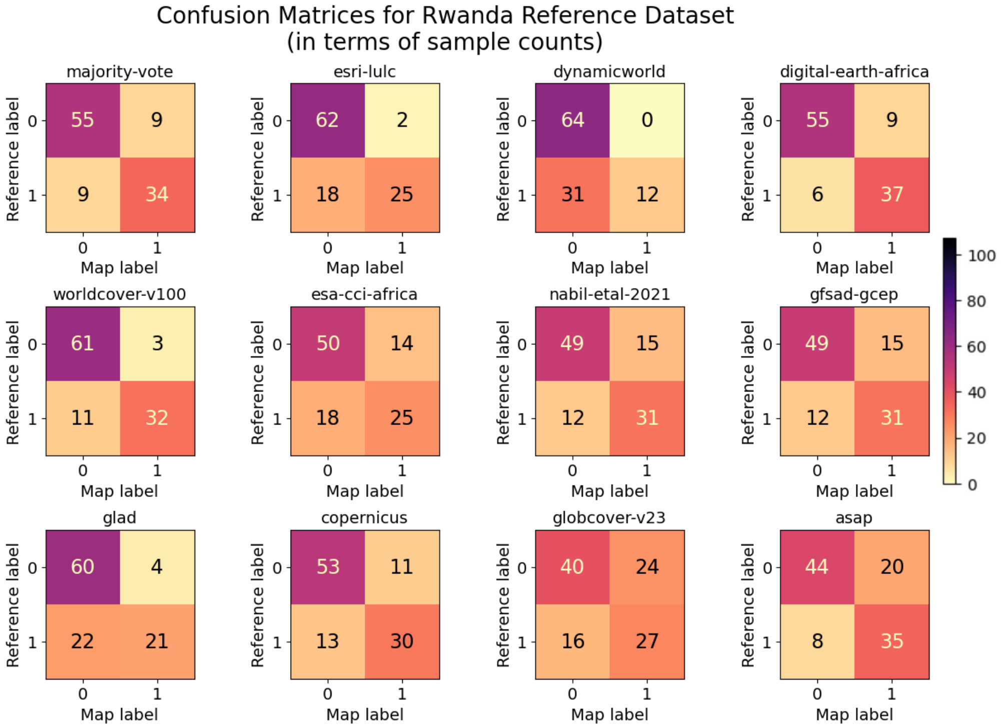

Figure 9. Unnormalized confusion matrix, expressed in terms of sample counts, for the Rwanda reference dataset.

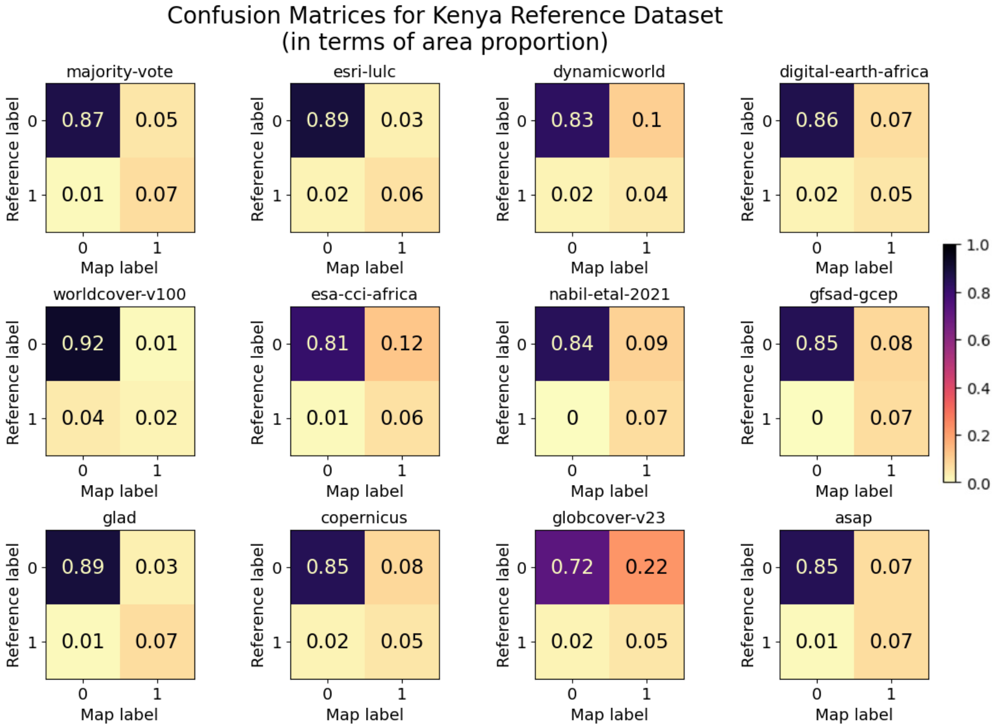

Figure 10. Population confusion matrix, expressed in terms of map area proportion, for the Kenya reference dataset.

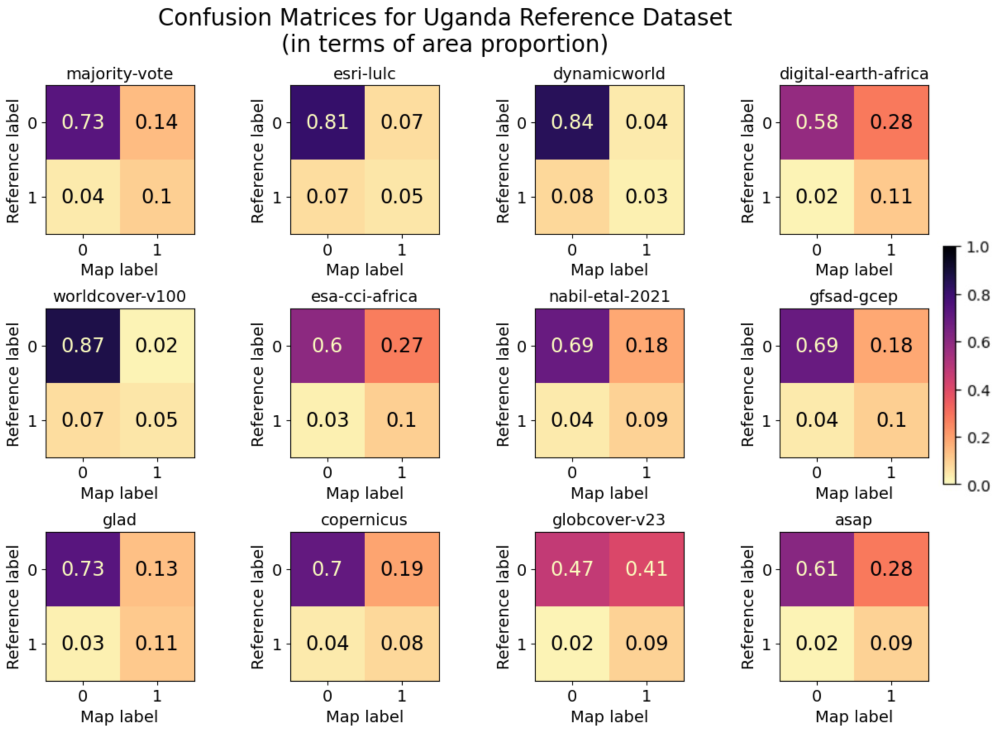

Figure 11. Population confusion matrix, expressed in terms of map area proportion, for the Uganda reference dataset.

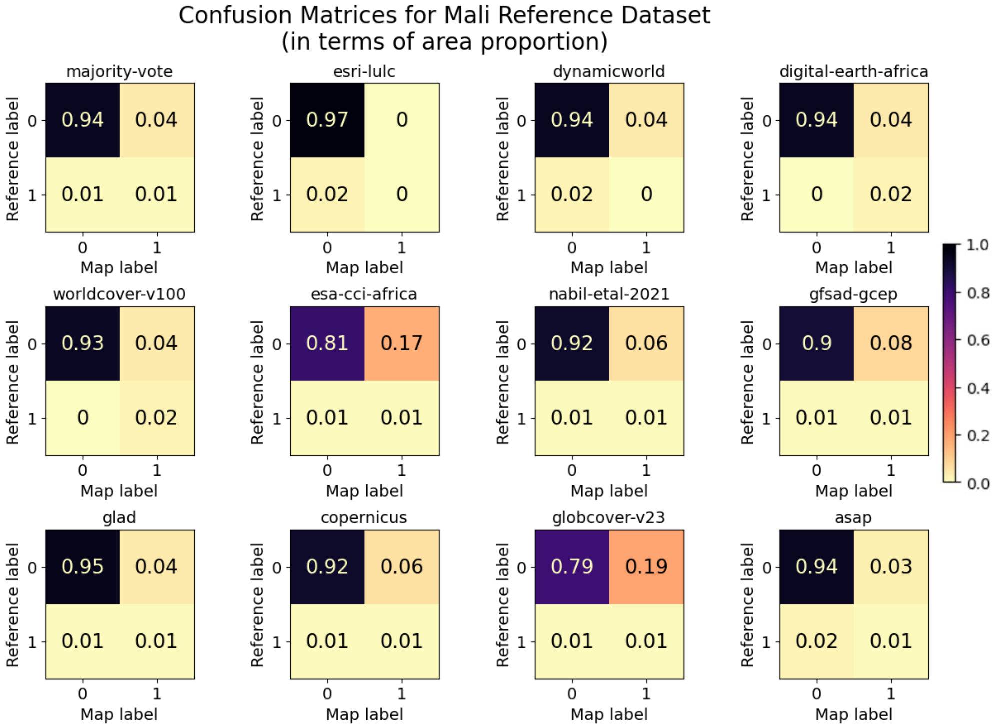

Figure 12. Population confusion matrix, expressed in terms of map area proportion, for the Mali reference dataset.

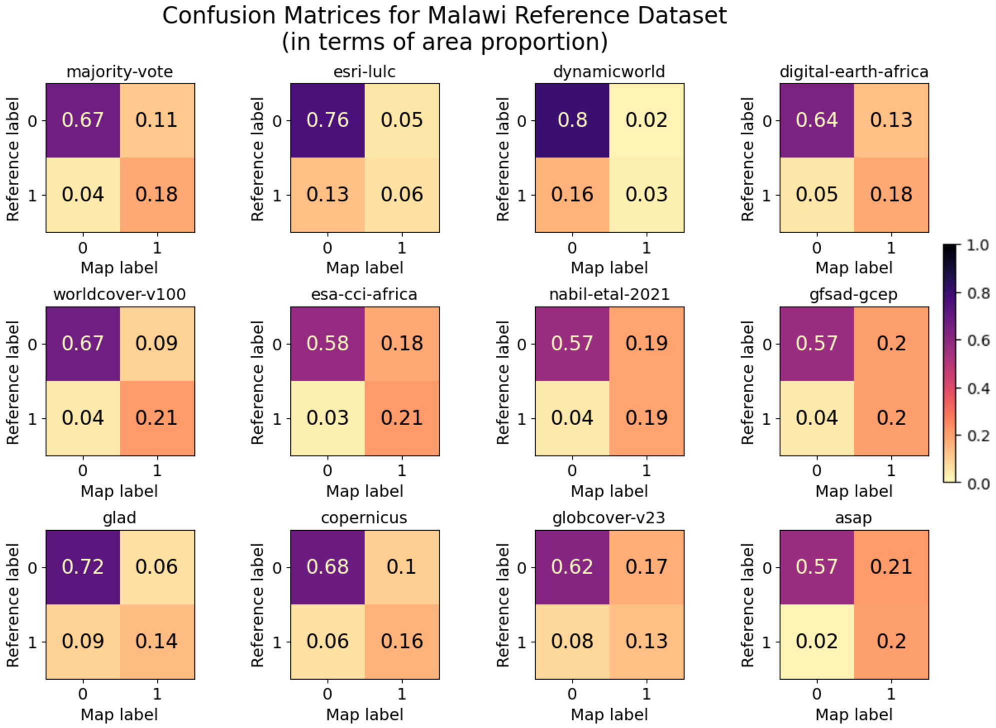

Figure 13. Population confusion matrix, expressed in terms of map area proportion, for the Malawi reference dataset.

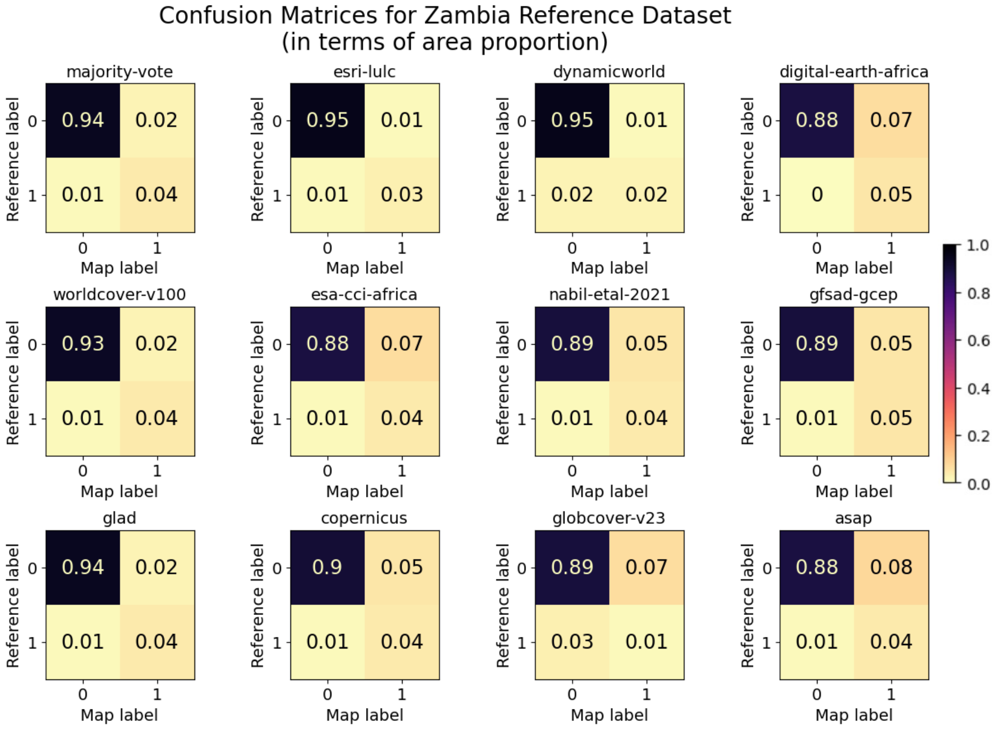

Figure 14. Population confusion matrix, expressed in terms of map area proportion, for the Zambia reference dataset.

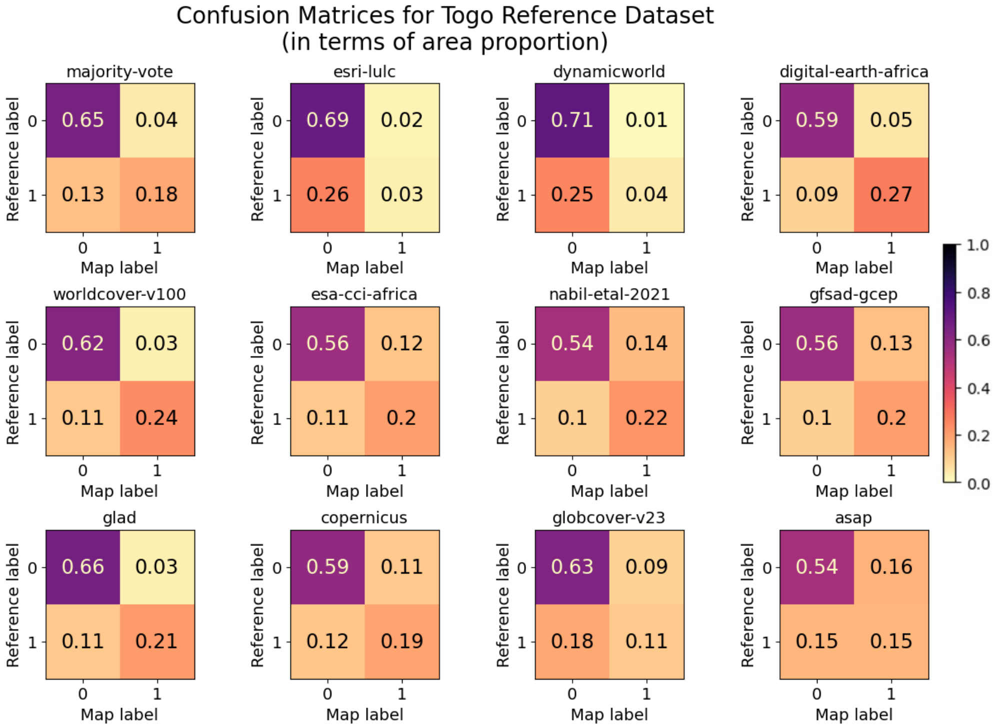

Figure 15. Population confusion matrix, expressed in terms of map area proportion, for the Togo reference dataset.

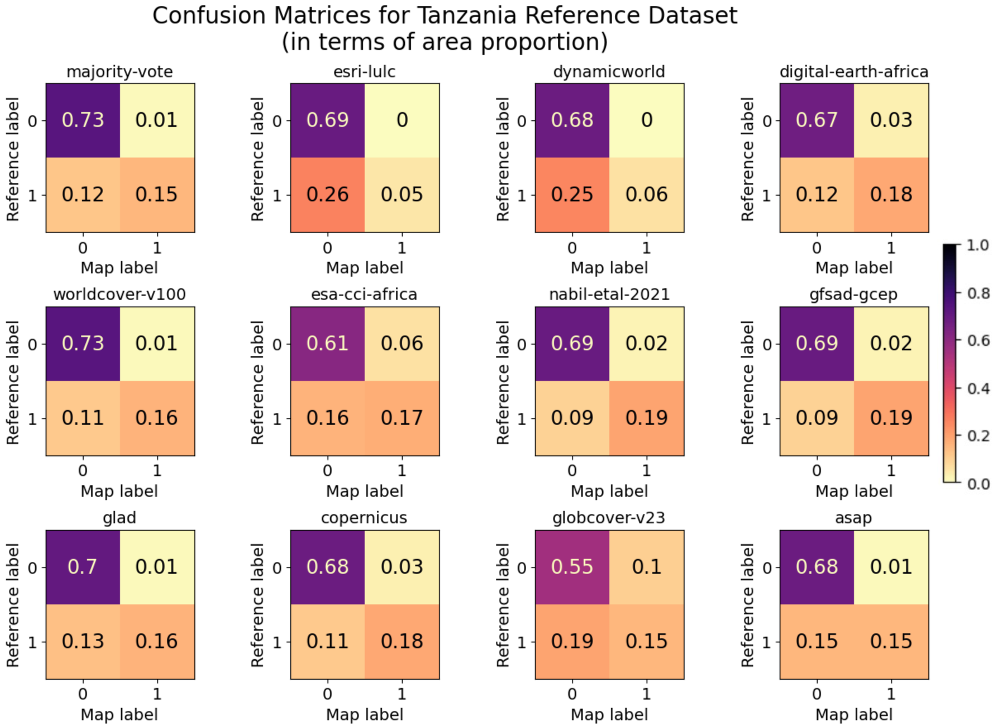

Figure 16. Population confusion matrix, expressed in terms of map area proportion, for the Tanzania reference dataset.

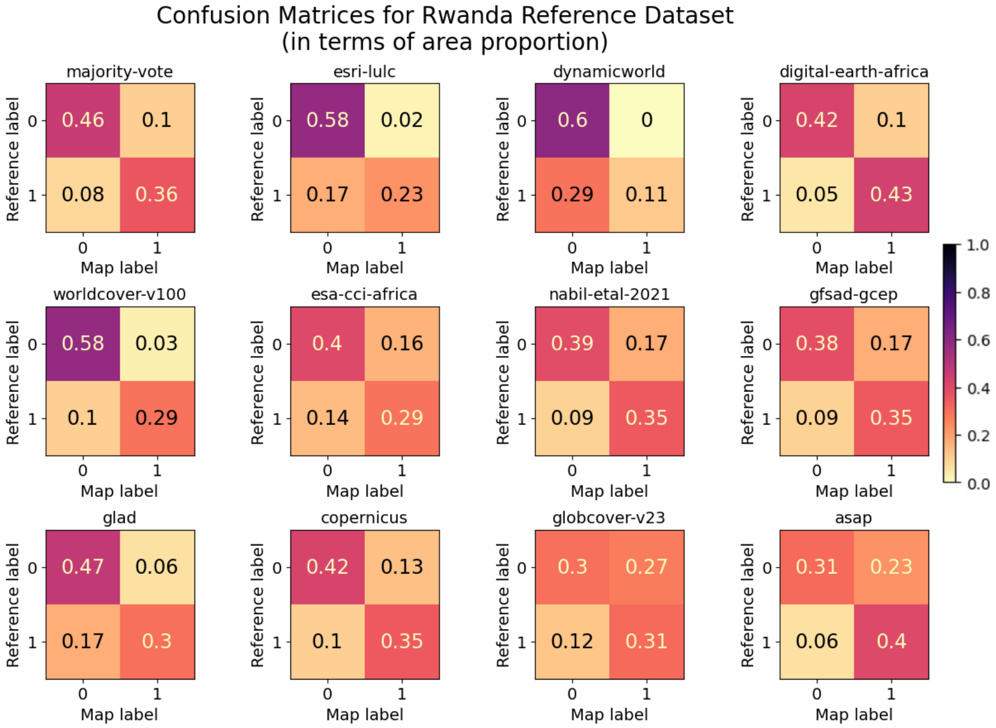

Figure 17. Population confusion matrix, expressed in terms of map area proportion, for the Rwanda reference dataset.
